# Pre-operative chemotherapy in early stage resectable non-small-cell lung cancer: a randomized feasibility study justifying a multicentre phase III trial

**DOI:** 10.1038/sj.bjc.6690241

**Published:** 1999-03

**Authors:** R H de Boer, I E Smith, U Pastorino, M E R O'Brien, F Ramage, S Ashley, P Goldstraw

**Affiliations:** Department of Medicine, Royal Marsden NHS Trust, Fulham Rd, London, SW3 6JJ, UK; Department of Thoracic Surgery, Royal Brompton Hospital, Sydney St, London, SW3 6JJ, UK

**Keywords:** non-small-cell lung cancer, pre-operative chemotherapy, mitomycin-C, vinblastine, cisplatin

## Abstract

Surgical resection offers the best chance for cure for early stage non-small-cell lung cancer (NSCLC, stage I, II, IIIA), but the 5-year survival rates are only moderate, with systemic relapse being the major cause of death. Pre-operative (neo-adjuvant) chemotherapy has shown promise in small trials restricted to stage IIIA patients. We believe similar trials are now appropriate in all stages of operable lung cancer. A feasibility study was performed in 22 patients with early stage (IB, II, IIIA) resectable NSCLC; randomized to either three cycles of chemotherapy [mitomycin-C 8 mg m^−2^, vinblastine 6 mg m^−2^ and cisplatin 50 mg m^−2^ (MVP)] followed by surgery (*n* = 11), or to surgery alone. Of 40 eligible patients, 22 agreed to participate (feasibility 55%) and all complied with the full treatment schedule. All symptomatic patients achieved either complete (50%) or partial (50%) relief of tumour-related symptoms with pre-operative chemotherapy. Fifty-five per cent achieved objective tumour response, and a further 27% minor tumour shrinkage; none had progressive disease. Partial pathological response was seen in 50%. No severe (WHO grade III–IV) toxicities occurred. No significant deterioration in quality of life was detected during chemotherapy. Pre-operative MVP chemotherapy is feasible in early stage NSCLC, and this study has now been initiated as a UK-wide Medical Research Council phase III trial. © 1999 Cancer Research Campaign


British Journal of Cancer (1999) 79(9/10), 1514–1518
© 1999 Cancer Research Campaign
Article no. bjoc.1998.0241

### Pre-operative chemotherapy in early stage resectable non-small-cell lung cancer: a randomized feasibility study justifying a multicentre phase III trial

**RH de Boer[1], IE Smith[1], U Pastorino[2], MER O'Brien[1], F Ramage[1], S Ashley[1] and P Goldstraw[2]**

[1]Department of Medicine, Royal Marsden NHS Trust, Fulham Rd, London SW3 6JJ; and [2]Department of Thoracic Surgery, Royal Brompton Hospital, Sydney St, London SW3 6JJ, UK

**Summary** Surgical resection offers the best chance for cure for early stage non-small-cell lung cancer (NSCLC, stage I, II, IIIA), but the 5-year survival rates are only moderate, with systemic relapse being the major cause of death. Pre-operative (neo-adjuvant) chemotherapy has shown promise in small trials restricted to stage IIIA patients. We believe similar trials are now appropriate in all stages of operable lung cancer. A feasibility study was performed in 22 patients with early stage (IB, II, IIIA) resectable NSCLC; randomized to either three cycles of chemotherapy [mitomycin-C 8 mg $m^{-2}$, vinblastine 6 mg $m^{-2}$ and cisplatin 50 mg $m^{-2}$ (MVP)] followed by surgery ($n = 11$), or to surgery alone. Of 40 eligible patients, 22 agreed to participate (feasibility 55%) and all complied with the full treatment schedule. All symptomatic patients achieved either complete (50%) or partial (50%) relief of tumour-related symptoms with pre-operative chemotherapy. Fifty-five per cent achieved objective tumour response, and a further 27% minor tumour shrinkage; none had progressive disease. Partial pathological response was seen in 50%. No severe (WHO grade III–IV) toxicities occurred. No significant deterioration in quality of life was detected during chemotherapy. Pre-operative MVP chemotherapy is feasible in early stage NSCLC, and this study has now been initiated as a UK-wide Medical Research Council phase III trial.

**Keywords:** non-small-cell lung cancer; pre-operative chemotherapy; mitomycin-C; vinblastine; cisplatin

Surgery offers the best chance of cure for patients with NSCLC, although no more than 20% are operable. Overall surgical 5-year survival rates are 55% for stage I, 25% for stage II, and 20% for stage III disease (Mountain, 1977); patients with completely resected T3 tumours without mediastinal node involvement have a slightly better outlook with approximately 40% 5-year survival (McCormack et al, 1987). These results can at best be described as moderate and it is likely that many surgical patients would accept the option of additional treatment to try to improve their outlook (Slevin et al, 1990).

Randomized trials of post-operative adjuvant chemotherapy in NSCLC have shown some prolongation of disease-free survival with cisplatin-containing schedules (Holmes et al, 1986; Lad et al, 1988; Niiranen et al, 1992). A meta-analysis of eight such trials has shown a 13% reduction in the risk of death, corresponding to an absolute survival benefit of around 5% at 5 years (Non-small Cell Lung Cancer Collaborative Group, 1995). A value judgement is required on the clinical benefit of such treatment, and it seems unlikely that significant further progress will be made with post-operative therapy using currently available drugs.

Pre-operative chemotherapy is currently being investigated in several tumour types including NSCLC, breast cancer and gastric cancer. In NSCLC, most pre-operative chemotherapy studies have been carried out in patients with stage IIIA disease. In non-randomized studies, response rates have ranged from 50% to 75% with occasional (10%) complete remissions; subsequent resectability rates of 65–90% and 3–5-year survival rates of 17–40% have been reported (Faber et al, 1989; Skarin et al, 1989; Weiden et al, 1991; Burkes et al, 1992; Strauss et al, 1992; Martini et al, 1993). The heterogeneity of entry criteria and the lack of data from randomized trials make these results difficult to interpret, but they make the important point that the majority of such patients appear to have chemosensitive disease prior to surgery. Two small randomized trials have compared surgery with or without pre-operative chemotherapy in patients with stage IIIA disease, each with similar and strikingly positive results in favour of chemotherapy. In the first study, median survival was 26 months for patients treated with pre-operative chemotherapy, compared with 8 months for patients treated with surgery alone (Rosell et al, 1994). The second study reported respective median survivals of 64 months vs 11 months (Roth et al, 1994).

These results, although encouraging, must be interpreted with caution because of the small number of patients randomized. They emphasize the need for a large multi-centre randomized trial, and we felt that this should involve *all* patients with operable lung cancer. Such an approach is novel and is associated with several uncertainties. These include the question of acceptability of pre-operative chemotherapy to both patient and doctor, the potential for unacceptable chemotherapy-induced toxicity prior to surgery, and the possibility of disease progression during chemotherapy.

We therefore decided to carry out a small randomized pilot feasibility study to address these issues, using three pre-operative cycles of MVP (mitomycin-C, vinblastine and cisplatin). This is a

*Received 26 June 1998*
*Revised 28 August 1998*
*Accepted 7 October 1998*

*Correspondence to:* I E Smith schedule which we have already shown to be active and well tolerated in advanced/metastatic NSCLC (Ellis et al, 1995). The primary aim of this pilot study was to assess feasibility of, and compliance with, pre-operative chemotherapy. The secondary aims were to assess response, symptom control, performance status, resectability and extent of surgery, side effects, and any technical difficulties that might arise with surgery following chemotherapy. We were also interested in assessing the quality of life of patients during the treatment schedule. We report our results here.

### MATERIALS AND METHODS

### Patients

Patients with previously untreated, histologically proven, NSCLC were invited to enter the study. Patient entry criteria were: a tumour considered to be resectable (i.e. stage I, II, or IIIA); a World Health Organization (WHO) performance status of 0–1; fitness for both the chemotherapy regimen and the proposed thoracic surgery and no history of prior malignancy. A haematological and biochemical screen was performed and renal function was assessed by creatinine clearance. The clearance was measured either by ethylenediamine tetra-acetic acid (EDTA) scan, or by the Cockcroft and Gault formula (Cockcroft and Gault, 1976). Radiological assessment of the tumour was required by both chest X-ray (CXR) and chest computed tomography (CT) scan. If there was radiological evidence of mediastinal node involvement ($N_2$), or chest wall plus hilar disease ($T_3N_1$), or if radiological staging was unclear, then mediastinoscopy and biopsy was performed. A bone scan was performed to rule out bony metastases. Patients accepting entry were required to sign a written consent form which had been approved by the ethics committee at each participating institution.

### Chemotherapy regimen

The chemotherapy regimen consisted of mitomycin-C 8 mg $m^{-2}$, vinblastine 6 mg $m^{-2}$ and cisplatin 50 mg $m^{-2}$ given every 3 weeks for three cycles (the mitomycin-C was only given in cycles 1 and 2). A strict hydration regime was followed consisting of 1 l of normal saline plus 10 mmol of $MgSO_4$ plus 40 mg of frusemide prior to chemotherapy, with a further 1.5 l of normal saline with a total of 15 mmol of $MgSO_4$ and 30 mmol of KCl as postchemotherapy hydration. Anti-emetic prophylaxis consisted of 8 mg of dexamethasone with 3 mg of granisetron given i.v. prior to chemotherapy with oral dexamethasone and oral domperidone to take home. Chemotherapy was only administered if adequate haematological recovery had occurred (neutrophil count > 1500 $mm^{-3}$ and platelet count > 100 000 $mm^{-3}$). If recovery had not occurred, treatment was delayed for a week. If there was a 2-week or more delay, than a 25% dose reduction was required. Creatinine clearance was calculated prior to each cycle, with the cisplatin dose adjusted if the clearance fell by more than 50% but remained greater than 60 ml $min^{-1}$. If the clearance fell by more than 50% of the starting value, or to less than 60 ml $min^{-1}$, then cisplatin was to be substituted by carboplatin; the dose determined by the Calvert formula (Calvert et al, 1989) with a desired area under curve (AUC) of 4.

### Assessment of toxicity and response

Chemotherapy-related toxicity was assessed and recorded after each cycle of treatment. Toxicity was measured using the WHO guidelines (Miller et al, 1981). Peri-operative and post-operative complications were documented by the surgical team. Patients were assessed both clinically and with CXR prior to each cycle of treatment. Symptomatic response and WHO performance status were recorded. Symptomatic response was graded as complete, partial, no change, or progressive according to the patient's own assessment. A chest CT scan was performed following the last cycle of chemotherapy to determine the radiological response. Responses were graded according to standard International Union Against Cancer criteria (Hayward et al, 1977). In addition, a minor response was recorded if there was a reduction in size of the index lesion of between 25% and 50% of the sum of the products of the maximum diameter and a perpendicular diameter. Any patients with evidence of clinical and/or radiological progression were to be removed from study and to undergo immediate thoracic surgery.

Pathological response was assessed by the on-site histopathologist. Pathological complete response was defined as absence of any tumour in the surgical specimen. Partial response was defined as the presence of only small residual foci of tumour cells, or the presence of significant areas of tumour necrosis throughout the surgical specimen. No change was defined as the presence of large areas of identifiable tumour cells in the surgical specimen with minimal evidence of tumour necrosis.

All patients receiving pre-operative chemotherapy were asked to complete European Organisation for Research and Treatment of Cancer (EORTC) QL Core 30 Quality of Life questionnaires prior to chemotherapy, after completion of the three cycles, and then at 3 months post-surgery. Patients in the surgery-only arm were also asked to complete EORTC QL Core 30 Quality of Life questionnaires prior to surgery, and at 1 and 3 months after surgery.

### RESULTS

Between July 1995 and May 1997, 96 patients were assessed for entry to this study; 56 were excluded because of poor performance status, comorbidity, previous cancers, or were found to be inoperable at mediastinoscopy. Of the 40 eligible patients, 22 (55%) consented to participate and 11 were randomized to each arm of the study. Of the remaining 18 patients, 17 gave as the reason for refusal to participate their concerns over further 'delay', and their preference for immediate surgery. One patient refused on the basis of a relative's 'bad' experience with chemotherapy. Patient demographics are shown in Table 1.

All 11 patients randomized to the chemotherapy arm received the planned three cycles of treatment. The median time between randomization and surgery for the chemotherapy-treated patients was 76.5 days (69–85 days), and for surgery-alone patients was 10 days (1–25 days).

One patient, initially assessed as operable and entered into the study, was considered on pre-operative evaluation to have been inoperable from presentation. Thus, the patient did not proceed to surgery. The patient had been randomized to, and received, three cycles of chemotherapy, and had a minor radiological response. This patient was included in the assessment of toxicity, performance status, radiological response and follow-up.

© Cancer Research Campaign 1999

**Table 1** Patient characteristics by treatment group

| Characteristic | Chemotherapy | Surgery alone |
|---|---|---|
| Sex | | |
| Male | 9 | 8 |
| Female | 2 | 3 |
| Median age (years) | 67 (44–77) | 60 (51–77) |
| TNM Stage | | |
| IA | 0 | 0 |
| IB | 9 | 10 |
| IIA | 0 | 0 |
| IIB | 1 | 1 |
| IIIA | 1 | 0 |
| Performance status (WHO) | | |
| 0 | 2 | 5 |
| 1 | 9 | 6 |
| Weight loss | | |
| Not significant | 10 | 11 |
| > 5% of pre-illness weight | 1 | 0 |
| Histology | | |
| Squamous cell carcinoma | 7 | 5 |
| Adenocarcinoma | 2 | 4 |
| Other (carcinoma/anaplastic/necrotic mixed) | 2 | 2 |

### Toxicity

Significant chemotherapy-related toxicity was minimal. There were no WHO grade 3 or grade 4 level toxicities. No admissions were required due to effects of the treatment and there were no chemotherapy dose reductions or dose delays. The most frequently noted toxicities were all of grade 1 level: nausea and vomiting (45% of patients), lethargy (36%), and alopecia and constipation (27%). Haematological toxicity did not appear to be a problem with no episodes of febrile neutropenia. Nephrotoxicity did not occur.

### Symptomatic responses; change in performance status

There was a good symptomatic response to MVP chemotherapy (Table 2). Of the 11 patients who were randomized to receive chemotherapy, three were asymptomatic at the start of treatment and remained that way throughout the three cycles. The other eight patients initially had a variety of symptoms including dyspnoea, cough, haemoptysis and pain. All eight had symptomatic improvement: four had a complete symptomatic response, and the other four had a partial response. No patient deteriorated.

Two of 11 patients experienced an improvement in performance status, both patients improving from PS 1 to PS 0. In both cases this improvement occurred between cycles 2 and 3. The other nine patients were stable throughout the three cycles. No deterioration in performance status occurred.

**Table 2** Responses in pre-operative chemotherapy patients ($n = 11$)

| Response | Symptomatic | Radiological |
|---|---|---|
| Complete response | 4 (36%) | 0 |
| Partial response | 4 (36%) | 6 (55%) |
| Minor response | 0 | 3 (28%) |
| Asymptomatic/no change | 3 (28%) | 2 (17%) |
| Progressive disease | 0 | 0 |

### Objective responses

Of the 11 patients receiving chemotherapy, six (55%) had a partial response (Table 2). The percentage reduction in the product of perpendicular diameters ranged from 50% to 82%. Three patients had a minor response (percentage reduction 29% to 46%), and two patients demonstrated no change in the size of their tumour. No patients had evidence of progressive disease during the treatment.

Of the six responding patients, three had squamous carcinoma, two had adenocarcinoma and one had undifferentiated carcinoma. All six were stage IB prior to treatment. The two patients who had no response to chemotherapy had squamous cell histology, one being stage IB and the other stage IIB.

### Surgical results

Of the 11 patients receiving immediate surgery, three had a single lobectomy, two had a bilobectomy, four had pneumonectomies and two had a lobectomy with resection of chest wall. Of the 10 patients who had surgery following chemotherapy, five had single lobectomies, two had bilobectomies, one had a pneumonectomy, one had a lobectomy with chest wall resection, and one underwent a sleeve resection of the main bronchus in addition to a lobectomy.

The surgery was tolerated well. There appeared to be no additional technical difficulties in the surgery performed on the patients who had received chemotherapy. There were no significant differences in the peri-operative and post-operative problems seen in the patients from each of the two arms. However, one patient in the chemotherapy arm did develop adult respiratory distress syndrome (ARDS) post-operatively and required a prolonged admission in intensive care before recovering. He had continuing problems with empyema post-discharge.

### Pathological findings

No patient had a pathological complete response. A partial pathological response was seen in five of 10 assessable patients (Table 3): three of these five were found to have minimal residual tumour, two having only small single nodules of tumour tissue remaining; the other two patients with a partial response had large areas of tumour necrosis.

### Quality of life

Quality of life analysis in patients receiving pre-operative chemotherapy showed that mean scores in four of the five func-

**Table 3** Pathological response post-chemotherapy ($n = 10$)

| | |
|---|---|
| Pathological complete response | 0 |
| Pathological partial response | |
| Minimal residual tumour | 3 |
| Large areas of necrosis | 2 |
| No apparent change | 5 |

*© Cancer Research Campaign 1999*

**Table 4** The average change in the mean scores of the functioning Quality of Life (QOL) scales between pre- and post-chemotherapy, and between pre-chemotherapy and 3 months post-surgery

| QOL scales | Change from pre- to post-chemotherapy | *P*-value* | Change from pre-chemotherapy to post-surgery | *P*-value* |
|---|---|---|---|---|
| Physical Functioning | No change | | –13.3 | 0.4 |
| Role Functioning | No change | | –25.0 | 0.2 |
| Emotional Functioning | +2.4 | 0.3 | –15.0 | 0.1 |
| Cognitive Functioning | +1.6 | 0.7 | –5.3 | 0.6 |
| Social Functioning | – 4.5 | 0.4 | –27.7 | 0.08 |

*Paired *t*-test.

tioning QOL scales had either improved slightly (Emotional and Cognitive Functioning), or remained stable (Physical and Role Functioning), following chemotherapy. None of the changes reached statistical significance. There was a small, but non-significant, decrease in Social Functioning (Table 4).

In the same patients, following surgery, there was a decrease in all the functioning QOL scale scores, with the largest decrease in the Social Functioning scale; again, none of these decreases reached significance (Table 4). Although an attempt was made to measure quality of life in the surgery-only patients, poor patient compliance with post-surgery questionnaires meant that meaningful analysis of this group was not possible.

### DISCUSSION

This study has confirmed that the administration of three cycles of MVP chemotherapy prior to surgery in patients with early stage NSCLC (IB and II as well as IIIA) is feasible, with 55% of eligible patients agreeing to participate. All patients randomized complied with the full treatment schedule. As previously shown in patients with stage IV NSCLC (Ellis et al, 1995), the MVP regimen proved to be easy to administer, and had minimal toxicity. There were no grade III/IV toxicities, and the most common toxicities were minor (grade I) nausea and lethargy. There were no dose reductions required for either haematological or nephro-toxicity. There were also no treatment delays, an important issue in a potentially curable tumour, with surgery proceeding as planned in all appropriate cases.

Although potential concerns have been voiced about delaying surgery during pre-operative chemotherapy, we found no evidence of disease progression during chemotherapy. Indeed, there was a suggestion that those patients receiving pre-operative chemotherapy required less extensive operations than those proceeding directly to surgery. This suggests that the benefit of chemotherapy-induced tumour regression outweighs the theoretical risk of tumour progression, a key point for confirmation in a phase III trial.

In this context, five patients showed pathological evidence of tumour regression, with two of the patients having only small residual nodules of tumour present in the surgical specimen. In this small series, no complete pathological remissions were seen, but these have been reported in other series. Martini et al (1993) found a 14% pathological complete response rate in 136 patients with N2 disease following two to three cycles of MVP chemotherapy. Rosell et al (1994) reported one complete response, and four cases with only residual microscopic tumour foci in 30 stage IIIA patients treated with three cycles of pre-operative mitomycin-C, ifosfamide and cisplatin.

A further useful benefit for pre-operative chemotherapy was the improvement in cancer-related symptoms prior to surgery. All patients who had symptoms at the time of randomization responded to chemotherapy, with half of them having a complete response. Two patients had improvement in their performance status, whilst the other nine remained stable. Patients receiving chemotherapy therefore arrived for surgery at least as fit as those proceeding directly to surgery, and sometimes fitter. We have also reported good symptomatic benefit from MVP in patients receiving chemotherapy for advanced, metastatic NSCLC (Ellis et al, 1995).

Quality of life data on the Physical, Role, Emotional, Cognitive and Social Functioning scales likewise suggested no significant deterioration during chemotherapy. It was of interest that surgery appeared to have a greater detrimental effect than chemotherapy, and this needs formal testing in a phase III trial.

A theoretical concern of pre-operative chemotherapy is increased peri-operative complications secondary to potential toxic effects of the drugs on pulmonary tissue. This particularly applies to the use of mitomycin-C, which has known pulmonary toxicity, but nearly always at larger cumulative doses than used here (Doyle et al, 1984; Linette et al, 1992). Although the numbers in this pilot study are too small to make definite conclusions, there did not appear to be a significant increase in peri-operative complications in the patients in the chemotherapy-treatment arm. Other randomized trials of preoperative chemotherapy have also not found a significant increase in surgical complications in the chemotherapy arm (Rosell et al, 1994; Roth et al, 1994).

In conclusion, the results of this small pilot randomized phase II study have shown that this approach is acceptable to more than 50% of eligible patients; the MVP chemotherapy regimen is well tolerated and brings patients to surgery with improved symptom control and, in some cases, improved performance status. We did not encounter disease progression or other adverse events during the chemotherapy or at surgery. The study therefore justifies a large multi-centre phase III trial in all patients with operable NSCLC to see whether the survival benefit suggested with pre-operative chemotherapy in the small randomised stage IIIA NSCLC trials can be confirmed. Such a trial is now underway under the auspices of the Medical Research Council Lung Group, and the data will be included in the currently running UK Big Lung Trial. Lung cancer specialists are invited to join.

### ACKNOWLEDGEMENTS

The authors would like to thank Cathy Ratcliffe for her assistance in data collection, and the resident medical and nursing staff who were involved in the treatment and care of the patients.

### REFERENCES

Burkes RL, Ginsberg RJ, Shepherd FA, Blackstein ME, Goldberg ME, Waters PF, Patterson GA, Todd T, Pearson FG, Cooper JD, Jones D and Lockwood G

*© Cancer Research Campaign 1999*

(1992) Induction chemotherapy with mitomycin, vindesine and cisplatin for stage III unresectable non-small-cell lung cancer: results of the Toronto phase II trial. *J Clin Oncol* **10**: 580–586

Calvert AH, Newell DR, Gumbrell LA, O'Reilly S, Burnell M, Boxall FE, Siddick ZH, Judson IR, Gore ME and Wiltshaw E (1989) Carboplatin dosage: prospective evaluation of a simple formula based on renal function. *J Clin Oncol* **7:** 1748–1756

Cockcroft DW and Gault MH (1976) Prediction of creatinine clearance from serum creatinine. *Nephron* **16**: 31–41

Doyle LA, Ihde DC, Carney DN, Bunn PA, Cohen MH, Matthews MJ, Puffenbarger R, Cordes RS and Minna JD (1984) Combination chemotherapy with doxorubicin and mitomycin C in non-small-cell bronchogenic carcinoma. Severe pulmonary toxicity from q3 weekly mitomycin C. *Am J Clin Oncol* **7**: 719–724

Ellis PA, Smith IE, Hardy JR, Nicolson MC, Talbot DC, Ashley SE and Priest K (1995) Symptom relief with MVP (mitomycin-C, vinblastine and cisplatin) chemotherapy in advanced non-small-cell lung cancer. *Br J Cancer* **71**: 366–370

Faber LP, Kittle CF, Warren WH, Bonomi PD, Taylor SG IV, Reddy S and Lee MS (1989) Preoperative chemotherapy and radiation for stage III non-small cell lung cancer. *Ann Thorac Surg* **47**: 669–677

Hayward JL, Carbone PP, Heuson J-C, Kumaoka S, Segaloff A and Rubens RD (1977) Assessment of response to therapy in advanced breast cancer. *Br J Cancer* **35:** 292–298

Holmes EC and Gail M (1986) Surgical adjuvant therapy for stage II and stage III adenocarcinoma and large cell undifferentiated carcinoma. *J Clin Oncol* **4**: 710–715

Lad T, Rubinstein L and Sadeghi A (1988) The benefit of adjuvant treatment for resected locally advanced non-small-cell lung cancer. *J Clin Oncol* **6**: 9–17

Linette DC, McGee KH and McFarland JA (1992) Mitomycin-induced pulmonary toxicity: case report and review of the literature. *Ann Pharmacother* **26**: 481–484

Martini N, Kris MG, Flehinger BJ, Gralla RJ, Bains MS, Burt ME, Heelan R, McCormack PM, Pisters KMW, Rigas JR, Rausch VW and Ginsberg RJ (1993) Preoperative chemotherapy for stage IIIA (N2) lung cancer: the Sloan-Kettering experience with 136 patients. *Ann Thorac Surg* **55**: 1365–1374

McCormack PM, Bains MS, Martini N, Burt ME and Kaiser LR (1987) Methods of skeletal reconstruction following resection of lung cancer invading the chest wall. *Surg Clin North Am* **67**: 979–986

Miller AB, Hoogstraten B, Staquet M and Winkler A (1981) Reporting results of cancer treatment. *Cancer* **47**: 207–214

Mountain CF (1977) Assessment of the role of surgery for control of lung cancer. *Ann Thorac Surg* **24**: 365–373

Niiranen A, Niitamo-Korhonen S, Kouri M, Assendelft A, Mattson K and Pyrhonen S (1992) Adjuvant chemotherapy after radical surgery for non-small-cell lung cancer: a randomised study. *J Clin Oncol* **10**: 1927–1932

Non-small Cell Lung Cancer Collaborative Group (1995) Chemotherapy in non-small cell lung cancer: a meta-analysis using updated data on individual patients from 52 randomised clinical trials. *Br Med J* **311**: 899–909

Rosell R, Gomez-Codina J, Camps C, Maestre J, Padille J, Canto A, Mate JL, Li S, Roig J, Olazabal A, Canela M, Ariza A, Skacel Z, Morera-Prat J and Abad A (1994) A randomised trial comparing preoperative chemotherapy plus surgery with surgery alone in patients with non-small-cell lung cancer. *N Engl J Med* **330**: 153–158

Roth JA, Fossella F, Komaki R, Ryan MB, Putnam JB Jr, Lee JS, Dhingra H, De Caro L, Chasen M, McGavran M, Atkinson EN and Hong WK (1994) A randomised trial comparing perioperative chemotherapy and surgery with surgery alone in resectable stage IIIA non-small-cell lung cancer. *J Natl Cancer Inst* **86**: 673–680

Skarin A, Jochelson M, Sheldon T, Malcolm A, Oliynyk P, Overholt R, Hunt M and Frei E (1989) Neoadjuvant chemotherapy in marginally resectable stage III M0 non-small cell lung cancer: long term follow up in 41 patients. *J Surg Oncol* **40**: 266–274

Slevin ML, Stubbs L, Plant HJ, Wilson P, Gregory WM, Armes PJ and Downer SM (1990) Attitudes to chemotherapy: comparing views of patients with cancer with those of doctors, nurses and general public. *Br Med J* **300**: 1458–1460

Strauss GM, Herndon JE, Sherman DD, Mathisen DJ, Carey RW, Choi NC, Rege VB, Modeas C and Green MR (1992) Neoadjuvant chemotherapy and radiotherapy followed by surgery in stage IIIA non-small-cell carcinoma of the lung: report of a Cancer and Leukemia Group B phase II study. *J Clin Oncol* **10**: 1237–1244

Weiden PL and Piantadosi S (1991) Preoperative chemotherapy (cisplatin and fluorouracil) and radiation therapy in stage III non-small-cell lung cancer: a phase II study of the Lung Cancer Study Group. *J Natl Cancer Inst* **83**: 266–273

 *© Cancer Research Campaign 1999*
